# A wirelessly controlled implantable LED system for deep brain optogenetic stimulation

**DOI:** 10.3389/fnint.2015.00008

**Published:** 2015-02-10

**Authors:** Mark A. Rossi, Vinson Go, Tracy Murphy, Quanhai Fu, James Morizio, Henry H. Yin

**Affiliations:** ^1^Department of Psychology and Neuroscience, Duke UniversityDurham, NC, USA; ^2^Triangle BioSystems InternationalDurham, NC, USA; ^3^Center for Cognitive Neuroscience, Duke UniversityDurham, NC, USA; ^4^Department of Neurobiology, Duke UniversityDurham, NC, USA

**Keywords:** channelrhodopsin, freely-behaving, wireless, optogenetics, direct pathway, striatonigral

## Abstract

In recent years optogenetics has rapidly become an essential technique in neuroscience. Its temporal and spatial specificity, combined with efficacy in manipulating neuronal activity, are especially useful in studying the behavior of awake behaving animals. Conventional optogenetics, however, requires the use of lasers and optic fibers, which can place considerable restrictions on behavior. Here we combined a wirelessly controlled interface and small implantable light-emitting diode (LED) that allows flexible and precise placement of light source to illuminate any brain area. We tested this wireless LED system *in vivo*, in transgenic mice expressing channelrhodopsin-2 in striatonigral neurons expressing D_1_-like dopamine receptors. In all mice tested, we were able to elicit movements reliably. The frequency of twitches induced by high power stimulation is proportional to the frequency of stimulation. At lower power, contraversive turning was observed. Moreover, the implanted LED remains effective over 50 days after surgery, demonstrating the long-term stability of the light source. Our results show that the wireless LED system can be used to manipulate neural activity chronically in behaving mice without impeding natural movements.

## Introduction

Recent advances in optogenetics have provided a method to selectively manipulate neural activity (Boyden et al., 2005; Zhang et al., 2006, 2007b; Han and Boyden, 2007). This method allows experimenters to excite or inhibit molecularly defined neuronal populations using genetically encoded light-gated ion channels or pumps. To study the behavior of awake behaving animals, the conventional method is to connect the chronic implant in the head to an external light source—commonly a diode laser—via fiber optic cables. Being physically connected to a laser, however, constrains natural movements. It greatly restricts the distance that animals can move from the light source, introducing torque to the cranial implant that can perturb free movement. It also limits the number of animals that can interact with one another during stimulation: e.g., two behaving rodents will become tangled if they are both connected to lasers with optic cables.

As neuroscience rapidly moves toward the goal of studying brain function under natural and ethologically realistic conditions, the above limitations present a major technical challenge. There is a strong demand for effective optical stimulation that does not rely on optic fibers. This requires both a local light source as well as a compact and lightweight power source. We developed a convenient system for wireless optogenetic stimulation using compact LEDs, with a number of advantages over recently developed systems (Wentz et al., 2011; Ameli et al., 2013; Kim et al., 2013). This system can also be easily expanded to permit simultaneous wireless recording and stimulation.

We tested the wireless stimulation system in the striatum, an input nucleus of the basal ganglia implicated in important behavioral functions including voluntary movement (DeLong, 1990; Graybiel, 1998; Yin and Knowlton, 2006; Rossi and Yin, 2011). We expressed channelrhodopsin-2 (ChR2) in striatal neurons that express D_1_-like dopamine receptors, i.e., neurons that give rise to the striatonigral (direct) pathway (Kravitz et al., 2012; Cui et al., 2013; Wall et al., 2013). In freely behaving mice, we used the wireless LED system to study the effect of striatonigral stimulation on behavior.

## Methods

### Subjects

All experiments were conducted in accordance with the National Institutes of Health guidelines regarding the care and use of animals and were approved by the Duke University Institutional Animal Care and Use Committee (Protocol Number: A027-14-02). For behavioral testing, male Ai32 mice expressing a floxed STOP cassette upstream of the ChR2(H134R)-EYFP gene (Madisen et al., 2012) were bred with dopamine D_1_ receptor Cre (D1-Cre) mice to yield D1-ChR2 mice that selectively expressed the light-gated cation channel, ChR2, in D1-expressing neurons (*n* = 3; aged 4–7 months). Controls were D1-Cre mice that did not express ChR2 (*n* = 3). For *in vivo* temperature measurements, a male C57BL/6 mouse aged 4 months was used.

### Construction of LED implant

The LED implant is a semi-rigid shank that consists of a thin and narrow printed circuit board (PCB), tiny surface mount LEDs at the narrower end of the PCB, and a small surface mount connector at the other wider end. This design allows the LEDs to be lowered directly into the desired brain region and illuminated without the use of optical fibers. Cree DA2432 Direct Attach bare chip LEDs were attached to the shank via a micro surface mount soldering technique using no lead solder (RoHS) and 40x optical zoom solder station. These LEDs have a typical wavelength of 465 nm. Typical forward voltage is 3.1 V at 20 mA with a maximum of 33 mW optical power output. The individual bare chip size is 320 × 240 × 140 μm, small enough so that many LEDs could be attached to the shank simultaneously and at precisely spaced locations. The implantable shank is 4 or 8 mm in length, 0.55 mm in width, and 0.035 mm in thickness. At the narrow end of the PCB, there are two surface mount pads for the anode and cathode of each LED, with pad dimensions of 0.508 × 0.178 mm, spaced 0.254 mm apart. Embedded copper routing traces run along the polyimide PCB and connect the surface mount pads through openings in the PCB mask to a surface mount connector at the wider end of the PCB. For the experiments, two LEDs were eutetically attached to the surface mount pads. Sterilized veterinarian's epoxy was then applied to the narrow end of the shank tip, to seal the LEDs and the openings of the polyimide mask along the flex PCB shank. After the epoxy cured for 24 h, the LED shank is then tested in saline solution for DC current leakage. This test procedure validates that zero current leakage occurs while the LED is on.

### Surgery

Mice were anesthetized with isoflurane (maintained at 1%) and a craniotomy was made above the anterior dorsal striatum. The LED shank was lowered into the brain targeting the final coordinates (in mm relative to bregma): AP +1.1; ML +1.4 to +2.4; DV −3.0. Two LEDs were oriented along the medio-lateral axis of the striatum facing posterior. The shank was secured with dental acrylic and skull screws. Mice were allowed to recover for 1 week before testing began. Following completion of behavioral tests, mice were deeply anesthetized and perfused with 4% PFA. Brains were post-fixed for 24 h, sliced with a Vibratome, and stained with DAPI or thionin to view the placement of the shank.

### Behavioral testing

On test days mice were connected to the wireless headstage and placed in an open chamber (7″ × 11.5″). Video was taken from directly above for off-line behavioral analysis. Mice were stimulated at for 30–60 s at 1, 10, and 20 Hz (5–50% duty cycle, 100% LED power). The order of stimulation was counterbalanced and mice were allowed to recover for 1–5 min between stimulations. To test stability of the LEDs, we tested two mice again 41 days after the initial test using the same parameters. Twitches were scored after the stimulation session and compared to the baseline behavior that occurred immediately before each stimulation. Turning was assessed in two mice using 20 Hz stimulation and 50% LED power.

### *In vivo* temperature measurements

Temperature change was measured using a Fluke temperature probe placed ~100 μm from the LED. A male c57BL/6J mouse was anesthetized with isoflurane and headfixed. The skull was opened over the striatum, and the LED and temperature probe assembly was lowered to a depth of 2 mm. Pulse trains lasted 60 s with varying duty cycles and LED power. Between pulse trains, the resting temperature was allowed to recover to baseline (~60 s) before the next pulse train was initiated. Temperature change was defined as the change between baseline temperature and the peak temperature measured during stimulation.

### Optical power measurements

Luminous flux was measured using a digital lux meter placed directly in front of the LED. The LEDs are tested for maximum optical power using a Thor Labs optical meter. During this test each LED is left on for 5 s at full brightness and the optical meter is placed within 2 mm of the LED surface to measure optical power. The forward current for the LED used is 5 mA. The maximum output current of the current driver is 20 mA (100%). The operation current of the LED is from 5 to 20 mA. The current used to determine radiant power of the LED ranges from 6.5 to 19.25 mA.

Optical power, P, was calculated as:

(1) $$\text{P}=\frac{\Phi \text{V}}{\eta },$$

where luminous flux, ϕV, was measured in lumens, and η is the product of Luminous Efficacy (683 lm/W) and the Spectral Wavelength Sensitivity Constant (0.09098 for λ = 470 nm).

## Results

### Chronically implantable wireless optogenetic stimulator

**Figure 1:**
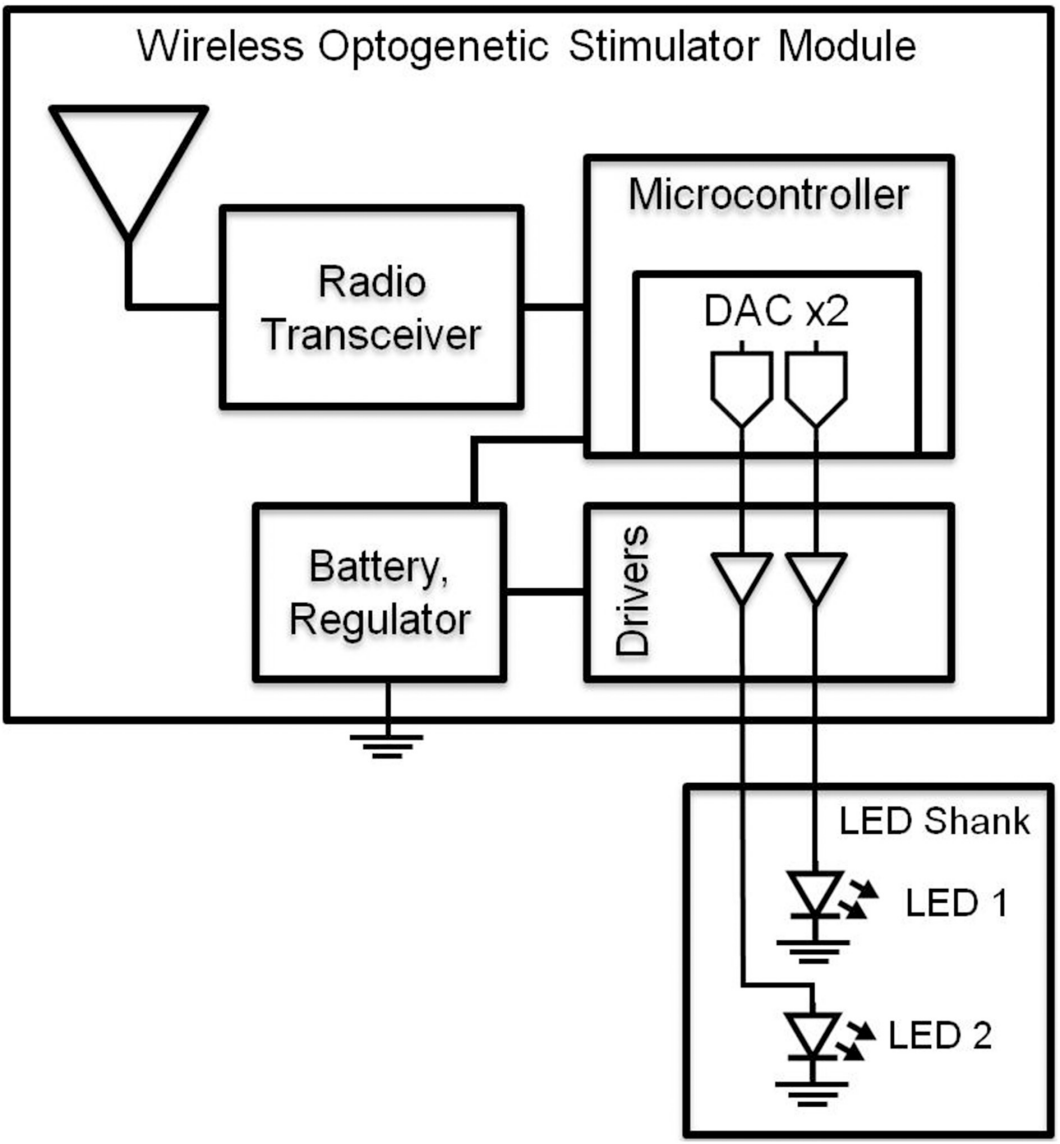
**Block diagram of wireless optogenetic stimulator**. A microcontroller containing two digital to analog converters (DAC) allows independent control of two blue LEDs.

**Figure 2:**
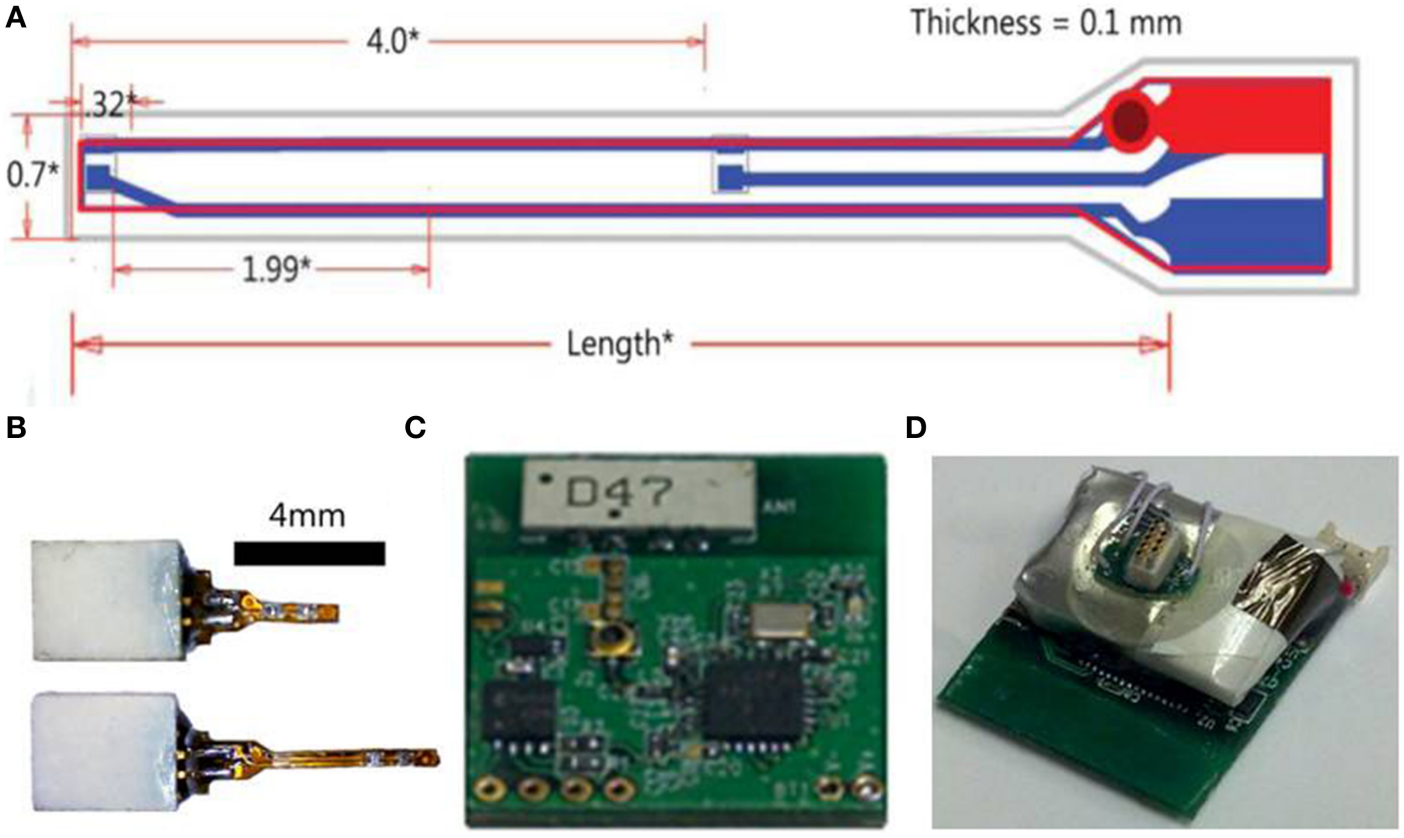
**Wireless optogenetic stimulation system with implantable LED**. **(A)** Illustration of LED shank (all measurements in mm). **(B)** LED implants with connectors. **(C)** Digital radio PCB. **(D)** Assembled opto-stimulator PCB.

To drive the LEDs wirelessly, we developed a multi-channel Gaussian frequency-shift keying (GFSK) transceiver PCB (Figures 1, 2) that receives radio signals (2.4–2.5 GHz; 250 kbps) from the transceiver located within a Universal Serial Bus (USB) dongle that can be connected to a nearby computer (range ~4 m). The microcontroller is a small 32-bit low-powered microprocessor that has built-in Flash and RAM memory and general-purpose digital inputs/outputs. The microcontroller is programmed by compiled C-language, and is stored in the Flash memory which maintains its memory even without being powered. The LED driver is an IC that outputs a constant current into the LED, which gives the LED steady or constant brightness. Our LED driver is controlled by a voltage input to control the amount of current being output into the LED, thus controlling the brightness.

The power source for the radio receiver and LEDs is connected to the PCB and the assembled opto-stimulator (headstage) is covered in epoxy to protect the electronics. The power source is a rechargeable lithium polymer battery that can be charged in ~20 min and lasts >2 h. The total weight of the headstage including the battery is 2.9 g. This lightweight design and long battery life makes this system ideal for experiments with freely moving small animals.

The software, OptoStim, has been developed to allow the user to control a variety of stimulation parameters in a simple graphical user interface. OptoStim is a LabView program that allows the user to control single pulse current and duration, train pattern (multiple pulses), stimulus pattern (multiple trains), remote headstage on/off switch, and manual pattern triggering for up to 16 channels independently. This affords the capability to independently stimulate up to 16 LEDs at different locations throughout the brain. The precise location and configuration of the LEDs can be easily adjusted depending on the experimental need.

### Optical power

**Figure 3:**
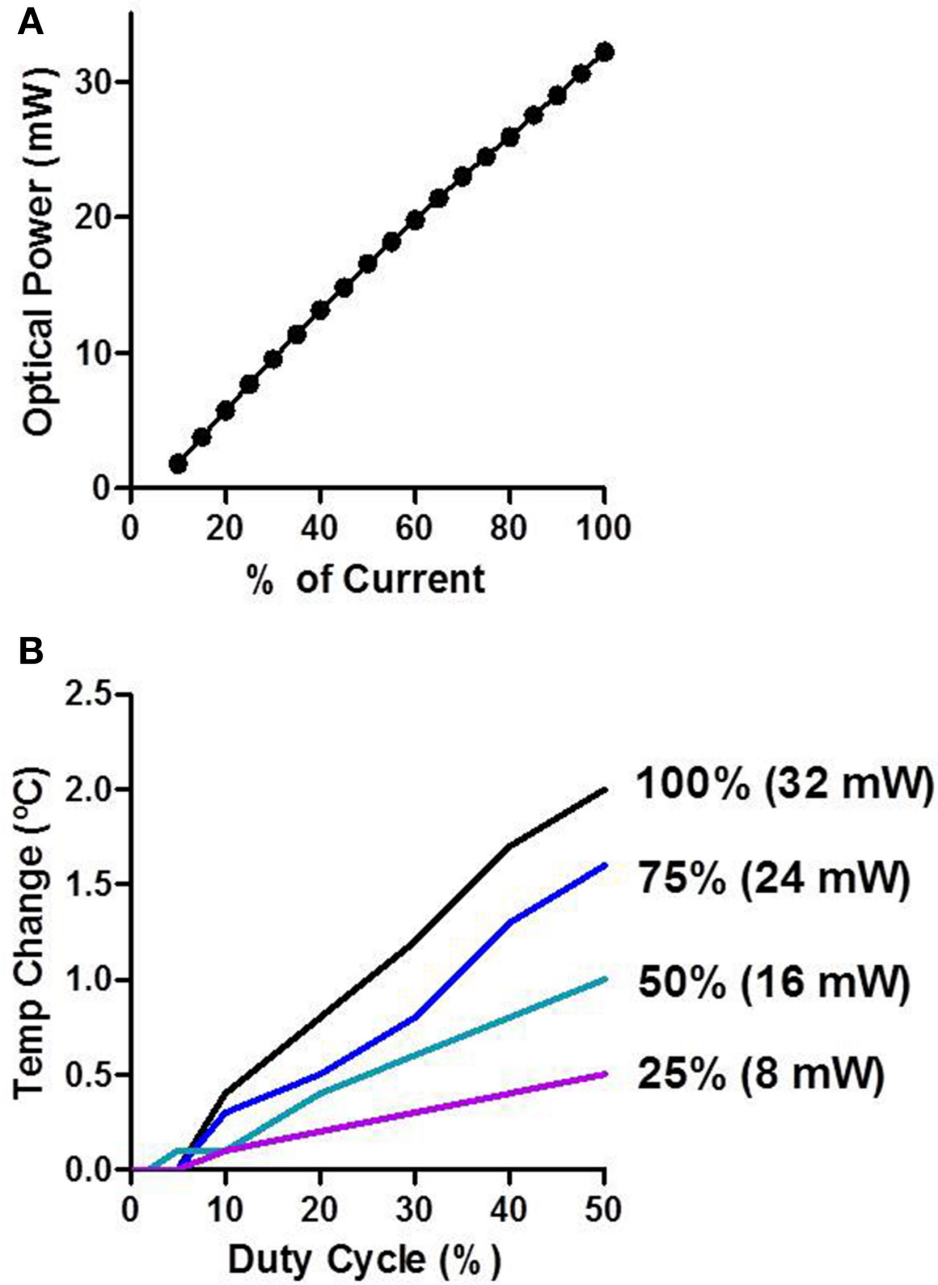
**Characterization of LED stimulation**. **(A)** Optical power increases linearly as a function of the input current. **(B)** Temperature change was measured *in vivo*. The temperature increases as a function of the duty cycle and the percent of input current.

We measured the optical power of the LEDs in air as a function of input current. There was a stable and linear relationship between LED input current and optical power produced (Figure 3A). The peak optical power produced from the LEDs was ~32 mW. We then measured how LED stimulation influenced the temperature of brain tissue surrounding the shank (Figure 3B). The temperature of neural tissue increased as a function of both stimulation duty cycle and optical power. Using common *in vivo* stimulation parameters (e.g., 20% duty cycle and 10 mW power), the temperature of the tissue was barely affected (~0.3°C).

### *In vivo* wireless optogenetic stimulation

**Figure 4:**
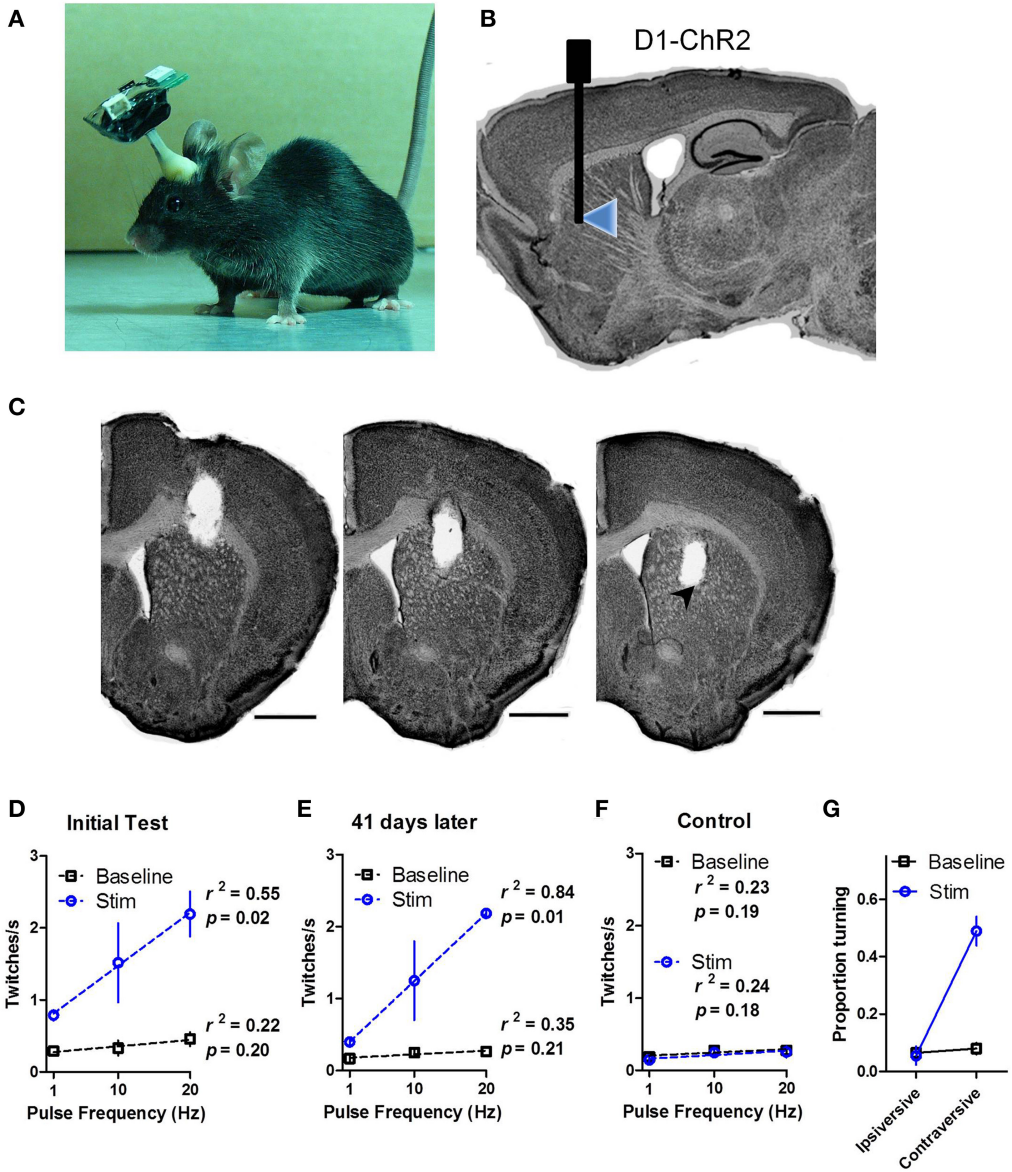
***In vivo* wireless stimulation of striatonigral neurons drives behavior**. **(A)** Photograph of a mouse with wireless headstage. **(B)** Schematic illustration of LED placement within the dorsal striatum of D1-ChR2 mice. **(C)** Representative serial coronal sections through the shank track. LED placement is indicated by arrowhead. Scale bars are 1 mm. **(D)** High power (32 mW) LED stimulation of striatonigral neurons induces twitching in freely behaving mice in a frequency dependent manner. **(E)** Behavioral response to LED stimulation is stable 41 days after the initial tests. **(F)** Control mice that lack opsin expression show no response to stimulation. The dotted lines are linear regression lines. **(G)** Proportion of time D1-ChR2 mice spent turning during low power (16 mW) stimulation. Values are mean ± s.e.m.

We demonstrated that the chronically implanted LEDs were able to elicit behavior reliably in freely moving mice. We implanted dual LED shanks in the dorsal striatum of D1-ChR2 transgenic mice that express ChR2 in direct pathway neurons or D1-Cre control mice (Figures 4A–C). We found that activation of direct pathway neurons in the striatum produced robust twitching behavior as well as spine bending and circling (Supporting Video 1). Twitches were scored offline by frame-by-frame video analysis. Stimulation induced twitching akin to dyskinesia in a frequency dependent manner [Figure 4D; Two-Way repeated measures ANOVA: main effect of Stimulation, *F*_(1, 8)_ = 36.83, *p* = 0.004; no main effect of Frequency, *F*_(2, 8)_ = 3.82, *p* = 0.07; no Interaction between the factors, *F*_(2, 8)_ = 2.37, *p* = 0.16]. Further analysis confirmed a linear relationship between the rate of twitching and the frequency of stimulation (linear regression, *r*^2^ = 0.55, *p* = 0.02 during stimulation; *r*^2^ = 0.22, *p* = 0.20 during baseline).

To test the stability of the LEDs we performed similar tests on two mice 41 days after the initial tests (Figure 4E). The twitching response was highly similar to the initial test (linear regression, *r*^2^ = 0.84, *p* = 0.01 during stimulation; *r*^2^ = 0.35, *p* = 0.21 during baseline), confirming the long-term functionality of the LEDs. D1-Cre control mice showed no response to stimulation (Figure 4F; *r*^2^ = 0.23, *p* = 0.19 during stimulation; *r*^2^ = 0.24, *p* = 0.18 during baseline).

Because we observed dyskinesia and robust twitching during high power stimulation, turning behavior was difficult to assess. During low power (16 mW) illumination, however, striatonigral activation reliably produced contraversive turning. The time spent turning in the contraversive direction was greatly increased during stimulation [Figure 4G; Two-Way repeated measures ANOVA: main effect of Stimulation, *F*_(1, 2)_ = 21.20, *p* = 0.04; main effect of Turn Direction, *F*_(1, 2)_ = 178.8, *p* = 0.0055; Interaction between Stimulation and Turn Direction, *F*_(1, 2)_ = 23.37, *p* = 0.04 driven by increased contraversive turning during stimulation relative to baseline, *p* < 0.05].

## Discussion

In recent years, optogenetic techniques utilizing fiber optics have been used extensively to investigate the function of intact neural circuits (Zhang et al., 2007a; Bernstein and Boyden, 2011; Stuber et al., 2011; Rossi et al., 2012, 2013). To reduce the constraint imposed by conventional optogenetic techniques on free behavior, we developed a chronically implantable LED stimulator that can target any brain region. Because the light source is located within the brain, this system makes it possible to remotely trigger complex stimulation patterns in freely behaving mice without the nuisance of optic fibers connecting the mouse to a laser. Because this system can remotely control multiple headstages independently, it is possible to perform experiments with multiple mice being stimulated simultaneously (e.g., social interaction or high-throughput behavioral analysis).

Using this system, we replicated previous results showing a bias toward contraversive turning during striatonigral stimulation (Tecuapetla et al., 2014). We were also able to observe for the first time a quantitative relationship between stimulation frequency and the rate of twitching (Figure 4, Supporting Video 1). Together with the observation that the firing rate of striatal output neurons can reflect movement velocity (Kim et al., 2014), this observation supports the recently proposed model that the striatonigral pathway is critical for modulation of the rate of transition in body configurations (Yin, 2014).

Other attempts to perform wireless optogenetic stimulation yielded systems that are either extremely difficult to construct and implement (Kim et al., 2013; McCall et al., 2013; Kwon et al., 2014; Lee et al., 2014) or use very large LEDs with limited spatial resolution (Iwai et al., 2011; Wentz et al., 2011). As summarized in Table 1, compared to these systems, the primary advantage of our design is its flexibility and the ease with which it can be implemented.

**Table 1:** **Comparison of our wireless stimulation system with other available wireless optogenetic stimulators**.

	**Our current OptoStim system**	**Competitor 1 (Wentz et al., 2011)**	**Competitor 2 (Kim et al., 2013)**	**Competitor 3 (Ameli et al., 2013)**
Battery life	2 h (20 min rechargeable)	None—RF Scavenging	None—RF Scavenging	None—Inductive power
Headstage size	14 × 17 × 5 mm	<1 cm^3^	~1 cm^3^ (est.)	15 × 25 × 17 mm
Headstage weight	2.9 g (including battery)	3 g	~2 g	7.4 g
LED size	240 × 320 × 140 μm	1 × 1 mm	50 × 50 × ~6.45 μm	Dimensions vary
LED wavelength	Blue 465.5 nm	Blue 470 nm	Various (including blue ~450 nm)	Various
Range	4 m	<1 m	Maximum unknown (tested 1–2 m)	>2 m (<7 cm power transmission)
LED location	Anywhere in the brain	Outside the brain (only for superficial brain regions)	Anywhere in the brain	Outside the brain. Light passed into brain via optic fiber
Time required for fabrication of implant; difficulty	<3 h; Easy	Exact details unknown 1 day for implant	~11–14 days for fabrication; Difficult (requires specialized materials science laboratory)	Exact details unknown 1 day for implant

*The specifications of our system are compared with optogenetic stimulation systems reported in recent publications (Wentz et al., 2011; Ameli et al., 2013; Kim et al., 2013; McCall et al., 2013)*.

Compared to the system described by Wentz and colleagues, our headstage is similar in size and weight. The advantage of our system is that the LED is small enough to target deep brain structures, whereas their system utilizes a large LED that must be placed outside the brain, thus limiting the stimulation to superficial regions. In our system, multiple LEDs can also be precisely placed on the implant to target different brain regions simultaneously, or different layers of layered structures such as the cerebral cortex.

The system described by Kim and colleagues, on the other hand, has a slightly smaller headstage, and allows for implantation of the LEDs in deep brain regions. Their system also incorporates a microelectrode for simultaneous electrophysiological recording and optogenetic stimulation. The main drawback of their system is that the fabrication and preparation is much more time consuming and requires a specialized materials science laboratory to implement. While the use of extremely small LEDs in their system can minimize damage, it also makes the implants difficult to fabricate. By comparison, all parts used in our wireless system are commercially available, and can be assembled by many neuroscience labs.

The system described by Ameli and colleagues has a much more massive headstage. While this system seems relatively easy to implement, at 7.4 g, this headstage will likely greatly impede the movement of mice. It appears more suitable for use in larger animals like rats. This system also uses an external, head-mounted LED that is coupled to an optic fiber in order to deliver the light to deep brain regions. For this reason, there is likely to be great power loss between the LED and the fiber, resulting in weak illumination within the brain.

A major advantage of our wireless system is its flexibility. It can easily be expanded to have more LEDs as well as other types of LEDs. The LEDs used in this study emitted blue (465.5 nm wavelength) light. This is useful for stimulating many excitatory channelrhodopsin variants including the cation channel, ChR2, as well as newly designed chloride conducting channels made from modified channelrhodopsin, i.e., ChloCs or iC1C2 (Berndt et al., 2014; Wietek et al., 2014). Thus, with blue LEDs, it is possible to excite or inhibit neural activity depending on the type of opsins expressed in the target neurons. It is also possible to attach similarly sized red or yellow LEDs to the shank that will function similarly to the blue LEDs.

Finally, the wireless stimulator assembly can also be combined with an existing wireless electrophysiology headstage (Fan et al., 2011; Barter et al., 2014; Kim et al., 2014) to produce a stimulation/recording headstage for simultaneous wireless recording and stimulation. Shanks can be made to target different regions in the brain based on stereotaxic coordinates. All this can in principle be accomplished using a single headstage light enough to be carried by a mouse or comparable small animals such as song birds. These flexible additions to the currently reported technique enable convenient study of ethologically realistic behavior in diverse species with wireless control, further expanding the capability of optogenetic tools in studying the neural substrates of behavior.

## Conflict of interest statement

Vinson Go, Tracy Murphy, Quanhai Fu, and James Morizio are employed by Triangle BioSystems International, a company that manufactures and sells the wireless LED system used in this paper. The authors declare that the research was conducted in the absence of any commercial or financial relationships that could be construed as a potential conflict of interest.
